# Phenolic Composition and Biological Properties of Wild and Commercial Dog Rose Fruits and Leaves

**DOI:** 10.3390/molecules25225272

**Published:** 2020-11-12

**Authors:** Milena Polumackanycz, Mateusz Kaszuba, Agnieszka Konopacka, Urszula Marzec-Wróblewska, Marek Wesolowski, Krzysztof Waleron, Adam Buciński, Agnieszka Viapiana

**Affiliations:** 1 Department of Analytical Chemistry, Medical University of Gdansk, Gen. J. Hallera 107, 80-416 Gdansk, Poland; milena.polumackanycz@gumed.edu.pl (M.P.); kaszuba.m997@gmail.com (M.K.); marek.wesolowski@gumed.edu.pl (M.W.); 2 Department of Pharmaceutical Microbiology, Medical University of Gdansk, Gen. J. Hallera 107, 80-416 Gdansk, Poland; agnieszka.konopacka@gumed.edu.pl (A.K.); krzysztof.waleron@gumed.edu.pl (K.W.); 3 Department of Biopharmacy, Nicolaus Copernicus University in Torun, Collegium Medicum in Bydgoszcz, 85-089 Bydgoszcz, Poland; u.marzec@cm.umk.pl (U.M.-W.); adam.bucinski@cm.umk.pl (A.B.)

**Keywords:** *Rosa canina* L., commercial/wild samples, fruits/leaves, biological activity, phenolic profile

## Abstract

*Rosa canina* L. (dog rose) is a rich source of phenolic compounds that offer great hope for the prevention of chronic human diseases. Herein, wild and commercial samples of dog rose were chemically characterized with respect to their phenolic composition by liquid chromatography coupled to diode array detection and electrospray ionization tandem mass spectrometry (LC-DAD-ESI/MS). Furthermore, in vitro antioxidant properties and antibacterial activity of dog rose fruits and leaves hydromethanolic extracts and infusions were also evaluated. The results revealed that wild and commercial fruits of dog rose are similar in terms of l(+)-ascorbic acid, total phenolics (TPC), total flavonoids (TFC) and total phenolic acids (TPAC) content, individual phenolic constituents and antioxidant activity. Moreover, the fruits had lower levels of phenolic compounds and also revealed lower biological activity than the leaves. On the other hands, the highest content of TPC, TFC, TPAC, individual phenolic constituents, DPPH (2,2-diphenyl-1-picrylhydrazyl) scavenging activity and FRAP (ferric reducing antioxidant power) were found in the leaf’s infusions. They were also the only ones to show antibacterial activity. Overall, these finding confirmed usefulness of *R. canina* L. leaves and fruits as a rich source of bioactive phenolic compounds with potential use in food, pharmaceutical, and cosmetic industries.

## 1. Introduction

Nowadays, there is an increasing trend for healthy, tasty and natural functional foods. Accordingly, food industry is focusing on developing new, natural and low processed food products with potential impact on the health and nutritional status of population. Medicinal plants emerge as alternative products, which are used not only in traditional medicine but also in a number of food and pharmaceutical products due to their nutritional value and bioactivity. Many of plant species of the Rosaceae family are considered to be of high importance because of their use in various food preparations. The *Rosa* genus comprises nearly 200 species that are widespread in temperate to subtropical habitats of Europe, Asia, North America and the Middle East [1]. The species *Rosa canina* L., also called dog rose, is a plant that is a rich source of biologically active compounds with chemoprevetion and antioxidant, antimutagenic and anticarcinogenic activities [2,3]. Hence, flowers, leaves, roots, branches and fruits of dog rose have been used for thousands of years for their medicinal benefits. Among those morphological parts, fruits and leaves are the most often used.

Fruits are generally used for prevention and treatment of some diseases such as common cold, gastrointestinal disorders, infections, various inflammatory diseases and chronic pains [1]. However, they also have antiobesity, antidiabetic, antinociceptive, antiulcerogenic and antiproliferate activity [4,5,6]. Fruits have been widely used in food, pharmaceutical and cosmetic industries as an ingredient of vitamin C tablets, food supplements, herbal remedies, and herbal teas. Consumption of fruits via traditional herbal teas, marmalades, jellies, jams, soups, food supplements, nectars, or wine is common in some European countries, especially in Germany, Poland, Portugal, Finland, Romania and Sweden [5,7]. Moreover, fruits have been applied as an ingredient of probiotic drinks and yoghurts. In Sweden and Germany some rose fruits are used to prepare a traditional Swedish soup “Nypon soppa” and German soup “Hagebuttern”, while in Turkey rose hips (the pseudo-fruits of Rosa species) are used as snacks [8]. Teas made from rose fruits have mild laxative and diuretic tendencies and help regulate the menstrual cycle, while teas from leaves and petals are soothing to the skin, and can help heal rashes and abrasions. Moreover, dry powders of dog rose fruits, which are marketed as food supplements reduce osteoarthritis symptoms in clinical trials [9].

The leaves of dog rose, in turn, have antioxidant and anti-inflammatory properties. Thus, in Turkish medicine they are used for the treatment of colds, flu, itching, eczema and cough [10]. They are the most commonly used in the form of tea. Leaves, in contract to the fruits have not been widely studied. To the best of our knowledge, there are scarce information on phenolic composition and antioxidant activity of dog rose leaves and antibacterial activity of leaves and fruits. On the other hand, some studies revealed differences in chemical composition between wild and commercial plant samples [11,12,13]. The differences can be linked to the origin of the sample (wild and commercial), and the type of preparation. Besides, in the case of commercial samples preserving, packing and storage time could affect their chemical composition. Hence, the objective of this study was to compare the phenolic composition and biological properties of water and hydromethanolic extracts prepared from the wild and commercial samples of dog rose fruits and leaves. Hydromethanolic extracts and infusions were prepared since the former are the most common way to obtain phenolic compounds enriched extracts, while the latter are the most frequently prepared for their common consumption.

## 2. Results and Discussion

### 2.1. Total Phenolic (TPC), Flavonoid (TFC), Phenolic Acids (TPAC) and l(+)-ascorbic Acid (ASA) Contents

TPC, TFC, TPAC and ASA were determined both in infusions and hydromethanolic extracts of wild and commercial dog rose samples. The results are summarized in Table 1. The infusions showed higher TPC, TFC and TPAC values than the hydromethanolic extracts. However, there are no remarkable differences between the content of l(+)-ascorbic acid in infusions and hydromethanolic extracts. TPC and TFC values concur with those obtained by Demir et al. [14], Nadpal et al. [15] and Tahinović et al. [16]. The content of l(+)-ascorbic acid is consistent with those values obtained by Demir et al. [14]—101.38 mg/100 g in hydromethanolic extracts, Fascella et al. [17]—513.95 mg/100 g in hydromethanolic extracts, and Nadpal et al. [15]—1.96–2.09 mg/g in water and 1.83–1.87 mg/g in methanolic extracts. Comparing two morphological parts of the dog rose, leaves (samples numbered 10–14) were richer in TPC, TFC, TPAC, ASA than the fruits in both extracts. Moreover, there were no significant differences between wild and commercial fruits, excluding the mean content of ASA in hydromethanolic extracts, 0.81 and 5.21 mg ASA/g DW, respectively, and TFC in infusions, 0.86 and 1.41 mg QE/g DW, respectively. The differences in vitamin C content between wild and commercial fruits may be due to various factors such as developmental stage/phase of the plant or enrich the plant material with vitamin C by producers.

Comparing the results obtained in this study to those found in the literature, Wenzing et al. [18] were found lower TPC in water extracts of commercial samples of rose hip with fruits and without fruits, 3.7 and 2.7 µg GAE/g, respectively, and higher TPC in methanolic extracts, 133 and 82.2 µg GAE/g, respectively. Koczka et al. [19] found lower TPC in water and ethanolic extracts of dog rose, over the range of 200–300 mg GAE/100 g and 400–500 mg GAE/100 g, respectively.

On the other hand, Dolek et al. [20] found higher TPC in harvested rose hips, 350–817 mg GAE/g fresh fruits. Slightly higher as well, TPC was reported by Fascella et al. [17], 6784.55 mg GAE/100 g in hydromethanolic extracts of dog rose, and Kerasioti et al. [21], 290 mg GAE/g dry weight (DW) of methanolic extracts. In the case of TFC, Kerasioti et al. [21] obtained higher values of TFC, 118.56 mg catechin/g DW, than those in this study. The TPAC in water extracts obtained by Tahinović et al. [16] concur with our findings, while TPAC in hydromethanolic extracts was higher, 5.46 mg caffeic acid/g. Regarding the dog rose leaves, Querghemmi et al. [22] found TPC in methanolic extracts on low level, 255 µg GAE/mg dry extract.

### 2.2. Individual Phenolic Compounds

The results for phenolic content in infusions and hydromethanolic extracts from wild and commercial *R. canina* L. samples are compiled in Table 2. Twelve phenolic compounds were identified and quantified by LC-DAD/ESI/MS. The differences were significant between the content of phytochemicals in infusions and hydromethanolic extracts. Overall, higher amounts of phenolic acids and flavonoids were found in infusions than in hydromethanolic extracts, while leaves were the richest in phenolic compounds. Wild and commercial fruits did not differ significantly in phenolic composition. Cinnamic acid and quercetin were more abundant in infusions of wild fruits, 11.87 and 7.31 µg/g DW, and commercial fruits, 10.71 and 6.21 µg/g DW, respectively. Moreover, chlorogenic and cinnamic acids predominated in infusions of wild leaves, 10.36 and 14.77 µg/g DW, respectively.

Infusions of samples 16 and 20 (commercial fruits), which contained also hibiscus were characterized by high cinnamic acid, rutin and quercetin contents. This may be due to the presence of other ingredients in the samples besides the dog rose fruits. It is well known that combining different herbs consisting of various bioactive constituents results in either synergistic or antagonistic biological effects. However, in the case of samples 16 and 20, combining the dog rose fruits and hibiscus did not improve the antioxidant potential of herbal tea products. It could be due to low amount of hibiscus or its bad quality. In addition, infusions of wild fruits no 7 and 8 were rich in cinnamic acid, while the samples 10 and 11 (leaves) were the richest in all phenolic constituents of analyzed dog rose samples. The highest concentration of rutin in hydromethanolic extracts of wild and commercial fruits was found to be 48.00 and 43.25 µg/g DW, respectively, while hydromethanolic extracts of wild leaves were richest in gallic and cinnamic acids, 9.10 and 6.28 µg/g DW, respectively. Rosmarinic and ferulic acids were found in the lowest amounts in infusions. It is noteworthy that some phenolic acids (syringic, *p*-coumaric, sinapic, ferulic and rosmarinic) were not detected in hydromathanolic extracts, however, they were found in alcoholic extracts of rose hips by Demir et al. [14], Nadpal et al. [15], Kerosioti et al. [21] and Elsmastaş et al. [23]. These variations may be due to different climatic and environmental conditions in which plants grow. Moreover, the maturity of the fruits can also contribute to the accumulation of phenolic compounds [23].

Overall, phenolic profile of wild and commercial samples of *R. canina* is consistent with earlier studies. Slight differences are only observed. The content of gallic acid in hydromethanolic extracts is similar to that found in cultivated Turkish dog rose [23], while in infusions is lower than that obtained by Demir et al. [14] and Nadpal et al. [15]. Moreover, the content of gallic acid and rutin determined by Kerasioti et al. [21] was similar to the values obtained in this study, 2.21 and 25.64 µg/g DW, respectively. However, Kerasioti et al. [21] found higher concentration of quercetin, 0.67 µg/g DW. Hosni et al. [24] found that ellagic acid and quercetin were the major compounds in all morphological parts of Tunisian dog rose. Otherwise, Querghemmi et al. [22] found rutin about five times lower (0.15 mg/100 g) in Tunisian dog rose leaves. Czyzowska et al. [25] revealed that chlorogenic acid is the most represented phenolic acid of the rose hip wines (40.91 mg/L), while ferulic and *p*-coumaric acids were detected in trace amounts. The changes in chemical composition of the dog rose samples can be due to either intrinsic and/or extrinsic factors, such as cultivation practice, storage conditions, type of soil, climatic factors and technological treatments [26]. Demir et al. [14] have also stressed out the importance on the chemical composition of Rosa species.

**Figure 1:**
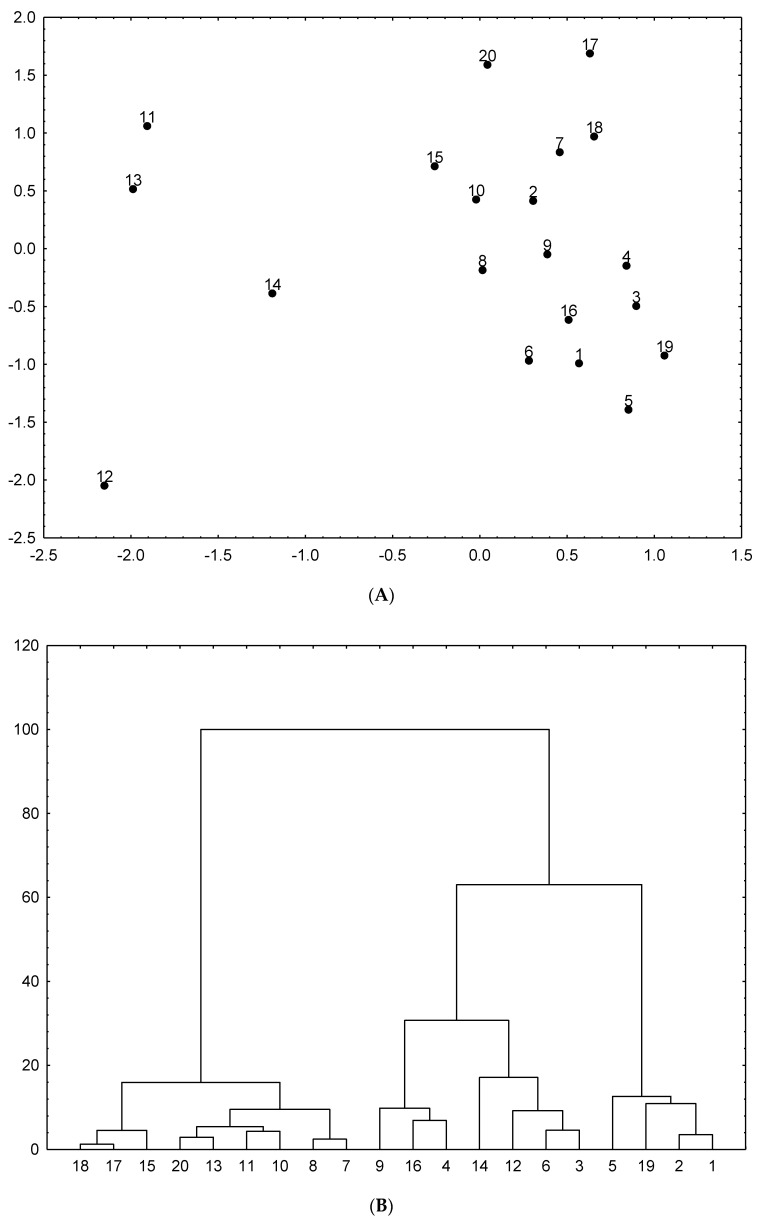
Principal component analysis scatterplot (**A**) and cluster analysis tree diagram (**B**) for wild and commercial samples of *R. canina* L.

To display similarity and dissimilarity between the samples of dog rose, principal component analysis (PCA) has been used. PCA calculations revealed that two first principal components (PCs) represents 53.96% of the total variance in the data set. The first PC1 with 42.46% of the total variance showed high loading (>−0.80) for cinnamic, syringic, protocatechuic and rosmarinic acids, and quercetin, rutin and TFC. The second PC2 with 11.50% of the total variance revealed high loading for FRAP values (0.74), TPC (0.75) and TPAC (0.80). PCA scores scatter plot (Figure 1A) highlighting differences between dog rose samples due to their chemical composition. Samples numbered 11, 12, 13 (leaves) and 14 (commercial fruits) can be found on the left side of PCA scatter plot. They have high content of phenolic compounds and high antioxidant activity. The wild and commercial fruits are located together on the right side of PCA plot. Similar relations were confirmed by the HCA as shown tree-diagram presented in Figure 1B.

### 2.3. Antioxidant Activity of R. Canina Samples

The results of DPPH and FRAP assays are compiled in Table 1. Higher antioxidant activity was obtained for the infusions, 14.56 mg TE/100 g DW and 98.66 mmol Fe^2+^/g DW, respectively, than for hydromethanolic extracts, 7.12 mg TE/100 g DW and 44.03 mmol Fe^2+^/g DW, respectively. It could be related to higher levels of the phenolic compounds in infusions. Other, non-phenolic compounds in hydromethanolic extracts, in turn, may exert some antagonistic effects, thus decreasing the antioxidant activity. Extracts prepared from the leaves showed the highest antioxidant activity, however, the DPPH and FRAP values for wild and commercial fruits of *R. canina* were on the same level. Infusions from samples 10, 11, 13, 14 (leaves) and hydromethanolic extract from sample 14 (leaves) were characterized by the highest values of DPPH and FRAP among all analyzed samples. The greater antioxidant activity of the leaves could be explained by the high polyphenolic content. Infusions of wild fruits no 2 and 4 and commercial fruits no. 15, 17, 18 and 20 were characterized by high values of DPPH and FRAP, while samples 5 (wild fruits) and 19 (commercial fruits) showed the lowest level of DPPH and FRAP values (below 34 mmol Fe^2+^/g DW). Among the hydromethanolic extract from fruits, the sample 2 (wild fruits) was characterized by the highest DPPH assay (above 9 mg TE/100 g), while the highest values of FRAP (above 60 mmol Fe^2+^/g) were determined for samples 2 and 7 (wild fruits).

Literature data revealed discrepancies between DPPH and FRAP values. Tahinović et al. [16] reported higher values of DPPH and lower values of FRAP for infusions and hydromethanolic extracts of cultivated *R. canina* fruits. Dolek et al. [20] determined lower FRAP values, over the range of 88–139 µmol TE/g for fresh fruit of rose hips. Otherwise, Koczka et al. [19] reported higher FRAP values for infusions and ethanolic extracts of dog rose, over the ranges of 200–250 and 350–400 mmol ascorbic acid/g DW, respectively. Demir et al. [14] determined the higher FRAP values (301.80 mmol TE/g DW) for hydromethanolic extracts of Turkish dog rose hips. The DPPH values obtained in this study cannot be compared with those obtained by Demir et al. [14] and Wenzing et al. [18] due to the difference in the calculation units. Similarly to phenolic compounds, antioxidant activity of dog rose also significantly depends on the environmental factors [27].

Correlation analysis revealed high positive relationships between the phenolic compounds contents and the antioxidant activity in infusions for the following pairs: TPC-FRAP (0.86), TPAC-FRAP (0.79), ASA-DPPH (0.75) and TFC-DPPH (0.68). No significant correlation was found between the TPC, TFC, TPAC and ASA, and the antioxidant activity of hydromethanolic extracts. A similar outcomes were also reported by Nadpal et al. [15], Wenzing et al. [18] and Querghemmi et al. [22]. This can be explained by the presence of other bioactive components with chemical structure different from phenolics, and by the interactions among these compounds [28]. In addition, individual phenolic compounds contribute to the antioxidant activity of dog rose extracts. For infusions, strong correlations were found between vanillic acid-DPPH (0.87), protocatechuic acid-DPPH (0.81), gallic acid-DPPH (0.72) and gallic acid-FRAP (0.73), while in hydromethanolic extracts seven positive correlations ranged from 0.48 (cinnamic acid-DPPH) to 0.63 (rutin-DPPH), and negative correlation between protocatechuic acid and DPPH (−0.52). The values of DPPH and FRAP were also correlated, moderate positive correlations were obtained, 0.57 and 0.68, for infusions and hydromethanolic extracts, respectively.

### 2.4. Antimicrobial Activity of Extracts

Preliminary microbiological studies against *S. aureus, E. coli and P. aeruginosa* strains revealed that infusions prepared from the wild fruits samples no 1, 2, 7 and hydromethanolic extracts prepared from samples no. 4 and 14 (wild fruits and leaves, respectively) did not exhibit antibacterial activity. Moreover, some infusions (samples no. 4, 5, 9, 18 and 19) and some hydromethanolic extracts (samples no. 1, 2, 5, 7, 11, 12, 13, 15 and 19) were weak exhibitors, and among these samples the best activities were obtained for leaves infusions against *S. aureus* with zone of inhibition in the range 11-22 mm. The others samples with microbiological activity were selected for the determination of MIC and MBC values in the screening test and the studies were expanded by adding another Gram-positive bacteria stains (two *S. aureus* MRSA, *S. epidermidis* and *S. pyogenes* strains). Based on the results (Table 3) infusions prepared from the leaves exhibited better activity against the Gram-positive bacteria strains. 

The activity of leaves infusions against Gram-negative bacteria strains was significantly weaker than expected, based on the diffusion test. Even samples for which growth inhibition zones with a diameter of 20–22 mm were obtained had significantly lower MIC values in comparison with analogical studies with Gram-positive bacteria strains. In the case of hydromethanolic extracts, the lowest MIC values were obtained for Gram-positive bacteria strains, especially *S. aureus* strain. The most active extract against all Gram-positive bacteria was found to be extract prepared from leaves (sample no. 10). Among the Gram-negative bacteria, hydromethanolic extracts exhibited the best activity against *E. coli* (MIC ranged between 32 and 128).

## 3. Materials and Methods

### 3.1. Plant Material

Twenty samples of dog rose (*Rosa canina* L.) compiled in Table 4 were analyzed. Six samples of fruits were purchased from pharmacy and local supermarkets, whereas nine samples of fruits and five of leaves were harvested during October-November 2018 in North part of Poland. Most of the commercial samples of *Rosa canina* L. were in a form of tea bags and only one of them was in a loose form. Moreover, two commercial samples contained also another herbs (samples no. 16 and 20). The dry samples were pulverized at 20 °C for 20 s in a water-cooled Knifetec 1095 grinder (Foss Tecator, Höganäs, Sweden) and until analysis were protected from light and humidity.

### 3.2. Standards and Reagents

2,2-diphenyl-1-picrylhydrazyl (DPPH reagent), 4-chloro-7-nitrobenzofurazan (NBD-Cl) and twelve analytical standards: rutin (RUT), quercetin (Q), and gallic (GA), protocatechuic (pCAT), vanillic (VA), chlorogenic (CGA), syringic (SA), *p*-coumaric (pCA), ferulic (FA), rosmarinic (RA), cinnamic (CNA) and sinapic (SNA) acids were purchased from Sigma-Aldrich (St. Louis, MO, USA). The purity of standards was higher than 98%. Aluminum chloride (AlCl_3_) was obtained from Across Organics (Morris Plains, NJ, USA), HPLC-grade acetonitrile (ACN) from J.T. Baker (Center Valley, PA, USA), while analytical and HPLC-grade methanol were purchased from POCh (Gliwice, Poland). Redistilled water was prepared by triple distillation of water in a Destamat ® bi-18 system (Heraeus Quarzglas, Hanau, Germany).

### 3.3. Sample Preparation

To prepare hydromethanolic extracts, a 1.0 g of a sample was sonicated with 4 mL of methanol-water mixture (80:20, *v*/*v*) for 20 min at 25 °C using an ultrasonic bath (Emag, Salach, Germany). The suspension was centrifuged in an EBA-20S centrifuge (Hettich, Tuttlingen, Germany) for 10 min at 8 000 rpm and the supernatant was transferred into a 20 mL volumetric flask. This procedure was repeated twice, and the extracts obtained were combined and diluted up to 20 mL with a mixture of methanol-water (80:20, *v*/*v*).

Water extracts (infusions) were prepared according to instructions found on the package of commercial products. A 1.0 g of a sample was mixed with 100 mL of boiling, distilled water and left to stand at room temperature under cover for 10 min. Then infusion was filtered through the Whatman filter paper no. 113 (Sigma-Aldrich, St. Louis, MO, USA).

### 3.4. Determination of Total Phenolic, Flavonoid and Phenolic Acid Contents

Total phenolic content (TPC) was determined using Folin-Ciocalteu (FC) reagent according to Singleton et al. [29]. A 0.3 mL of extract was mixed with 0.2 mL of FC reagent and after 2 min, 2 mL of 7% Na_2_CO_3_ (*w*/*v*) was added. The sample was incubated in a dark at room temperature for 60 min and next, the absorbance was measured at 760 nm. Gallic acid was used as a standard and the results were given as gallic acid equivalent in mg/g dry weight of the sample (mg GAE/g DW).

To determine total flavonoid content (TFC) pharmacopoeial method was used [30]. A 1.2 mL of the diluted extract was mixed with 0.1 mL of 5% AlCl_3_ (*w*/*v*) solution and with 1.4 mL of acetic acid and methanol (1:19) mixture. After 30 min of incubation in a dark, the absorbance was measured at 430 nm. The results were given as quercetin equivalent in mg/g dry weight of the sample (mg QE/g DW).

Total phenolic acid content (TPAC) was determined using Arnov’s reagent [31]. To 1.4 mL of extract, 0.2 mL Arnov’a reagent, 0.2 mL hydrochloric acid and 0.2 mL sodium hydroxide were added and then the absorbance was measured at 490 nm. The results were given as coffee acid equivalent in µg/g dry weight of the sample (µg CAE/g DW).

### 3.5. Determination of l(+)-ascorbic Acid

Method developed by Abdelmagged et al. [32] was used to determine of l(+)-ascorbic acid content (ASA). A 0.3 mL of extract was mixed with 0.2 mL of sodium hydroxide, 0.2 mL of 4-chloro-7-nitrobenzofurazane (NBD-Cl) and 1.4 mL of acetone-water mixture (50:50%, *v*/*v*). Then the sample was incubated in a dark and after 30 min the absorbance was measured at 582 nm. The obtained results were given as ascorbic acid in mg/g dry weight of the sample (mg ASA/g DW).

### 3.6. Individual Phenolic Compounds Determination

Liquid chromatography coupled to diode array detection and electrospray ionization tandem mass spectrometry (LC-DAD/ESI/MS) (Shimadzu, Kyoto, Japan) was used for quantitation of phenolic compounds. LCMS system consisting of two LC pumps (LC-20AD), a DGU-20A3 degasser, an autosampler set at 12 °C (SIL-20AC), a column oven set at 40 °C (CTO-20AC), a diode array detector (SPD-M20A) and a mass spectrometry detector (LCMS-2010EV, QoQ system: Q-array—Octapole—Quadrupole mass analyzer, Shimadzu, Kyoto, Japan). The detection was performed using 280, 320 and 370 nm as preferred wavelength for DAD. The parameters of electrospray ionization were as follows: detector voltage 1.5 kV, heat block temperature 270 °C, CDL (curve desolvation line) temperature 250 °C and nebulizing gas flow 1.5 L/min. Chromatographic runs were performed using a Hypersil Gold C18 column (250 × 4.6 mm, 5 µm particles) equipped with a guard column (4 × 4.6 mm) (both from Thermo Scientific, Mainz, Germany). The samples were analyzed at 40 °C using solvent A (0.5% acetic acid in water) and solvent B (0.5% acetic acid in acetonitrile-water mixture (50:50, *v/v*)) as mobile phase with the following gradient: 0 min: 5% B; 20 min: 10% B; 40 min: 15% B; 72 min: 20% B; 140 min: 43% B; 180 min: 100% B; 184 min: 100% B; 198 min: 5% B; 200 min: 5% B. The solvent flow rate was 0.2 mL/min.

The phenolic compounds were identified by comparing their retention time, and UV-Vis and mass spectra with those for standard compounds. The validation parameters for quantitation of phenolic compounds are listed in Table 5. Calibration curves prepared for twelve phenolic compounds were linear and reproducible with correlation coefficient (R^2^) > 0.993. The results were expressed as µg of phenolic compound per g dry weight of the sample (µg/g DW). The limits of detection (LOD) and quantification (LOQ) were determined as the injection concentration provided peak heights 3- and 10-fold the signal-to-noise ratio (s/n). The LOD and LOQ were expressed as µg/mL. The lowest values of LOD and LOQ were obtained for *p*-coumaric acid, 2.65 µg/mL and 6.98 µg/mL, respectively, while the highest for chlorogenic acid, 5.66 µg/mL and 16.89 µg/mL, respectively.

### 3.7. Antioxidant Activity

DPPH test was performed using method of Tuberoso et al. [33]. A 2 mL of extract was mixed with 1.6 mL of DPPH reagent. After 10 min of incubation, absorbance was measured at 517 nm. The obtained results were expressed in mg Trolox equivalent per 100 g dry weight of the sample (mg TE/100 g DW).

FRAP assay was carried out using the method of Benzie and Strain [34]. Sample containing 200 µL of the extract was mixed with 2.25 mL of FRAP reagent and after 30 min of incubation in the dark, absorbance was measured at 593 nm. The results expressed in mmol ferrous ion equivalents per g dry weight of the sample (mmol Fe^2+^/g DW).

### 3.8. Antibacterial Activity

The antimicrobial activity of extracts was examined according to the procedures and guidelines of EUCAST (European Committee on Antimicrobial Susceptibility Testing) and CLSI (Institute of Clinical and Laboratory Standards).

#### 3.8.1. Bacterial Strains and Growth Conditions

Antibacterial activity was tested using Gram-positive strains: *Staphylococcus aureus* ATCC 6538, *Staphylococcus aureus* MRSA 18532, *Staphylococcus aureus* MRSA 43300, *Staphylococcus epidermidis* ATCC 14990, group A β-hemolytic *Streptococcus* (from the own collection at the Department of Pharmaceutical Microbiology), Gram-negative strains: *Escherichia coli* ATCC 8739, *Pseudomonas aeruginosae* ATCC 9027. Group A β-hemolytic *Streptococcus* was cultured in Brain Heart Infusion (BHI) broth (Becton Dickinson, Franklin Lakes, NJ, USA) with 5% bovine serum (Biomed, Lublin, Poland) or Mueller-Hinton (MH) agar with 5% sheep blood, and incubated in a 5% CO_2_ atmosphere at 37 °C. Another stains were grown in Mueller-Hinton broth and MH agar (Becton Dickinson, Franklin Lakes, NJ, USA) at 37 °C.

#### 3.8.2. Agar Well Diffusion Assay

A preliminary study of the antimicrobial activity of extracts was carried out using an agar well diffusion test. The following strains were used: *Staphylococcus aureus* ATCC 6538, *Escherichia coli* ATCC 8739, *Pseudomonas aeruginosae* ATCC 9027. Molten cooled MH agar (25 mL) was inoculated with 1 mL a suspension of appropriate bacterium at a density of 106 CFU/mL, then was poured in the sterile Petri dish with cylinders set. Upon solidification of the agar, the cylinders were removed to give wells in diameter 7 mm. Then, 0.5 mL of each extract (130 mg) were added to respective wells. After the pre-incubation for 1 h at room temperature, the plates were incubated for 24 h at 37 °C to obtain bacterial growth. After incubation, the diameter of the zone of growth inhibition was measured. Then Minimal Inhibitory Concentration (MIC) and Minimal Bactericidal Concentration (MBC) values were determined for the extracts that showed the growth inhibition zone in agar well diffusion method.

#### 3.8.3. MIC and MBC Assays

The MIC was determined by the broth microdilution technique. After filling each well with 50 μL of broth, dry test samples were dissolved in water to a final concentration of approx. 1000 mg/mL. These solutions of tested extracts (50 μL) were added to the first well of each microtiter line. Dilution in geometric progression was conducted by transferring the dilution (50 μL) from the first to the twelfth well. An aliquot (50 μL) was discarded from the latter well. A microbial suspension (50 μL) was added to each well. The final concentration of the extracts used to the antimicrobial activity ranged from 0.25 to 256 mg/mL. Tests were incubated in the appropriate conditions described above at 37 °C for 48 h. Because the extracts were colorful, determination of the MIC values was difficult. In addition, 100 μL of suspension from each well was inoculated on an agar plate to control bacterial viability. After incubation 48 h the presence or absence of bacteria growth was verified. The concentrations at which a reduction of 50–90% of bacteria was achieved were taken as the MIC with a reduction of 99.9% of the bacteria as MBC.

### 3.9. Statistical Analysis

The analyses were carried out in triplicate. The results were expressed as arithmetic mean ± standard deviation (SD). One way analysis of variance (ANOVA) was applied for statistical data analysis, followed by Duncan test. A Pearson’s correlation coefficients were calculated between the individual phenolic constituents, TPC, TFC, TPAC and ASA, and the antioxidant activity. The calculations were performed at a 95% confidence level. Principal component analysis (PCA) and hierarchical cluster analysis (HCA) using the Ward’s clustering were carried out in order to evaluate the ability of phenolic compounds in combination with antioxidant properties to classify wild and commercial dog rose samples. The analyses were performed using a Statistica 10 software (StatSoft Inc., Tulsa, OK, USA).

## 4. Conclusions

The study on phenolic profile and biological activity of infusions and hydromethanolic extracts prepared from wild and commercials fruits and leaves of *Rosa canina* L. revealed that the infusions are richer in phenolic composition and have higher antioxidant potential than the hydromethanolic extracts. Quercetin and rutin were found in the higher amount in infusions, whereas chlorogenic, gallic and cinnamic acids in hydromethanolic extracts. Moreover, infusions and hydromethanolic extracts of leaves were characterized by the highest phenolic compounds content and biological activity, while no significant differences were found between wild and commercial fruits of dog rose. This study also revealed that the phenolics and flavonoids and antioxidant activity are highly correlated, while PCA and HCA indicated significant differences in the chemical profile and biological activity between extracts prepared from leaves and fruits. Thus, the obtained data suggest that leaves and fruits of *R. canina* L. contain significant amounts of phenolic compounds which positively affect the human health. Especially dog rose leaves should be used as a potential source of antioxidant agents. Moreover, data obtained in this study confirm that not only dog rose fruits but also its leaves can be used in household products and also in pharmaceutical and food industry as a rich source of bioactive compounds.

## Figures and Tables

**Table 1:** Total phenolic (TPC), flavonoid (TFC), phenolic acid (TPAC), l(+) ascorbic acid (ASA) contents and DPPH and FRAP assays in the samples of *Rosa canina* L.

	TPC	TFC	TPAC	ASA	DPPH	FRAP
Water Extracts	[mg GAE/g]	[mg QE/g]	[µg CAE/g]	[mg ASA /g]	[mg TE/100g]	[mmol Fe^2+^/g]
1.	54.82 ± 1.72 ^f^	0.76 ± 0.07 ^bc^	1.45 ± 0.10 ^a^	1.81 ± 0.23 ^d^	5.63 ± 0.03 ^d^	47.20 ± 5.82 ^c^
2.	104.86 ± 2.63 ^m^	0.99 ± 0.01 ^d^	5.45 ± 0.10 ^h^	4.15 ± 0.14 ^gh^	20.37 ± 0.05 ^l^	94.9 ± 3.39 ^h^
3.	44.59 ± 3.71 ^c^	0.63 ± 0.01 ^ab^	3.05 ± 0.11 ^d^	1.96 ± 0.10 ^d^	1.95 ± 0.04 ^a^	61.32 ± 1.74 ^d^
4.	54.04 ± 3.62 ^f^	0.84 ± 0.01 ^c^	3.24 ± 0.14 ^de^	1.96 ± 0.22 ^d^	21.05 ± 0.05 ^m^	89.42 ± 2.22 ^g^
5.	49.9 ± 8.46 ^d^	0.66 ± 0.02 ^ab^	2.32 ± 0.23 ^c^	0.97 ± 0.16 ^b^	4.43 ± 0.08 ^c^	27.75 ± 3.51 ^a^
6.	52.6 ± 3.18 ^e^	0.52 ± 0.01 ^a^	1.93 ± 0.17 ^b^	2.72 ± 0.07 ^e^	2.71 ± 0.02 ^b^	89.65 ± 5.60 ^g^
7.	157.45 ± 4.52 ^r^	0.62 ± 0.01 ^ab^	10.12 ± 0.12 ^k^	1.69 ± 0.11 ^d^	11.58 ± 0.03 ^i^	149.54 ± 4.41 ^p^
8.	112.19 ± 3.61 ^n^	0.51 ± 0.03 ^a^	5.62 ± 0.17 ^h^	0.77 ± 0.06 ^b^	11.71 ± 0.04 ^i^	97.44 ± 5.18 ^i^
9.	82.24 ± 3.65 ^i^	0.55 ± 0.02 ^a^	3.71 ± 0.08 ^f^	0.46 ± 0.13 ^a^	9.29 ± 0.02 ^g^	102.07 ± 3.80 ^k^
10.	96.05 ± 3.23 ^k^	2.54 ± 0.87 ^g^	8.45 ± 1.65 ^i^	3.65 ± 0.56 ^f^	27.53 ± 3.82 ^n^	99.67 ± 2.49 ^j^
11.	144.38 ± 2.43 ^o^	3.18 ± 0.23 ^i^	10.74 ± 1.22 ^l^	5.08 ± 0.62 ^j^	35.64 ± 3.94 ^r^	168.07 ± 7.32 ^s^
12.	56.62 ± 3.28 ^g^	4.85 ± 0.58 ^j^	5.26 ± 0.65 ^h^	4.26 ± 0.76 ^h^	17.87 ± 1.42 ^k^	103.22 ± 4.78 ^k^
13.	177.05 ± 4.35 ^s^	2.96 ± 0.74 ^h^	4.69 ± 1.64 ^g^	4.67 ± 0.95 ^i^	30.85 ± 2.72 ^p^	154.11 ± 1.84 ^r^
14.	76.19 ± 2.97 ^h^	3.12 ± 0.76 ^i^	3.65 ± 0.78 ^ef^	3.98 ± 1.01 ^g^	29.51 ± 2.85 ^o^	87.99 ± 3.82 ^f^
15.	103.43 ± 2.88 ^l^	1.03 ± 0.03 ^d^	10.68 ± 0.14 ^l^	2.81 ± 0.06 ^e^	8.57 ± 0.03 ^f^	122.6 ± 2.61 ^m^
16.	35.22 ± 1.40 ^b^	1.58 ± 0.05 ^f^	3.24 ± 0.10 ^de^	1.38 ± 0.09 ^c^	9.55 ± 0.09 ^g^	68.96 ± 1.41 ^e^
17.	101.27 ± 1.90 ^l^	1.59 ± 0.03 ^f^	8.66 ± 0.15 ^ij^	2.87 ± 0.29 ^e^	10.83 ± 0.11 ^h^	141.44 ± 1.49 ^o^
18.	89.89 ± 2.72 ^j^	1.51 ± 0.03 ^f^	8.97 ± 0.21 ^j^	3.01 ± 0.16 ^e^	9.21 ± 0.03 ^g^	105.24 ± 1.16 ^l^
19.	33.63 ± 0.84 ^a^	1.28 ± 0.02 ^e^	1.12 ± 0.10 ^a^	0.70 ± 0.09 ^ab^	6.50 ± 0.07 ^e^	33.61 ± 3.25 ^b^
20.	145.18 ± 7.23 ^p^	1.08 ± 0.05 ^d^	11.01 ± 0.16 ^l^	3.57 ± 0.08 ^f^	16.5 ± 0.16 ^j^	129.04 ± 2.50 ^n^
Hydromethanolic extracts						
1.	21.5 ± 0.10 ^a^	0.15 ± 0.01 ^j^	0.68 ± 0.11 ^d^	1.2 ± 0.02 ^h^	8.02 ± 0.06 ^gh^	39.44 ± 7.11 ^i^
2.	48.97 ± 1.03 ^k^	0.16 ± 0.01 ^j^	0.43 ± 0.04 ^ab^	0.88 ± 0.07 ^fg^	9.23 ± 3.03 ^ij^	64.40 ± 11.14 ^m^
3.	24.6 ± 0.13 ^bc^	0.13 ± 0.03 ^i^	0.73 ± 0.10 ^e^	0.58 ± 0.06 ^c^	5.89 ± 1.18 ^d^	34.64 ± 0.48 ^f^
4.	22.6 ± 0.24 ^ab^	0.09 ± 0.01 ^cd^	0.54 ± 0.14 ^c^	0.79 ± 0.09 ^gh^	4.36 ± 0.68 ^ab^	32.74 ± 1.05 ^d^
5.	21.4 ± 0.65 ^a^	0.07 ± 0.02 ^ab^	0.46 ± 0.08 ^b^	0.65 ± 0.04 ^de^	8.44 ± 0.83 ^h^	34.23 ± 5.51 ^ef^
6.	22.8 ± 0.12 ^ab^	0.10 ± 0.03 ^efgh^	0.64 ± 0.16 ^d^	0.96 ± 0.06 ^g^	7.49 ±2.04 ^efg^	47.07 ± 3.28 ^j^
7.	26.5 ± 0.32 ^cde^	0.11 ± 0.04 ^ghi^	0.84 ± 0.19 ^g^	1.51 ± 0.04 ^j^	7.28 ± 1.77 ^ef^	69.81 ± 2.07 ^n^
8.	33.7 ± 0.03 ^h^	0.10 ± 0.02 ^defg^	0.98 ± 0.17 ^h^	0.71 ± 0.08 ^ef^	7.17 ± 1.49 ^e^	54.43 ± 4.87 ^k^
9.	54.5 ± 0.14 ^l^	0.09 ± 0.01 ^cde^	0.73 ± 0.14 ^e^	0.35 ± 0.06 ^a^	7.95 ± 1.04 ^fgh^	37.42 ± 4.63 ^h^
10.	25.3 ± 0.22 ^cd^	0.27 ± 0.05 ^l^	1.56 ± 0.17 ^l^	0.48 ± 0.02 ^b^	6.39 ± 1.07 ^d^	18.41 ± 2.41 ^a^
11.	30.2 ± 0.09 ^fg^	0.51 ± 0.010 ^o^	0.86 ± 0.08 ^g^	5.35 ± 1.09 ^n^	7.21 ± 1.03 ^e^	58.34 ± 4.51 ^l^
12.	26.0 ± 0.23 ^cde^	0.31 ± 0.03 ^m^	1.44 ± 0.13 ^k^	6.06 ± 0.66 ^p^	9.45 ± 2.19 ^j^	76.77 ± 4.15 ^o^
13.	26.9 ± 0.24 ^de^	0.46 ± 0.22 ^n^	1.38 ± 0.16 ^j^	2.80 ± 0.42 ^l^	8.65 ± 1.01 ^hi^	53.57 ± 8.11 ^k^
14.	22.5 ± 0.19 ^ab^	0.25 ± 0.03 ^k^	1.05 ± 0.14 ^i^	8.79 ± 0.32 ^s^	13.11 ± 3.10 ^k^	81.50 ± 4.81 ^p^
15.	27.3 ± 0.61 ^de^	0.06 ± 0.01 ^a^	0.66 ± 0.05 ^d^	2.20 ± 0.02 ^k^	8.61 ± 0.91 ^hi^	24.93 ± 5.43 ^c^
16.	28.1 ± 0.12 ^ef^	0.08 ± 0.02 ^cd^	0.54 ± 0.08 ^c^	4.27 ± 0.41 ^m^	5.92 ± 1.07 ^d^	22.63 ± 1.51 ^b^
17.	44.0 ± 0.12 ^j^	0.12 ± 0.02 ^hi^	0.84 ± 0.12 ^g^	8.29 ± 4.34 ^s^	3.66 ± 0.38 ^a^	36.24 ± 5.19 ^g^
18.	32.1 ± 0.03 ^gh^	0.11 ± 0.04 ^fgh^	0.83 ± 0.20 ^g^	5.72 ± 2.62 ^o^	3.78 ± 0.11 ^a^	23.58 ± 1.71 ^b^
19.	26.5 ± 0.41 ^cde^	0.08 ± 0.01 ^bc^	0.42 ± 0.10 ^a^	1.31 ± 0.56 ^i^	4.64 ± 0.76 ^bc^	33.17 ± 8.72 ^de^
20.	41.7 ± 0.34 ^i^	0.09 ± 0.03 ^cdef^	0.77 ± 0.29 ^f^	6.45 ± 1.70 ^r^	5.10 ± 1.07 ^c^	37.31 ± 9.90 ^gh^

Means followed by the same letter within a column indicate no significant difference (*p* < 0.05) in Duncan test.

**Table 2:** The content of phenolic acids and flavonoids (µg/g DW) in the samples of *Rosa canina* L.

	GA	CGA	CNA	VA	SA	pCA	pCAT	SNA	FA	RA	Q	RUT
Water extracts												
1.	0.73 ± 0.24 ^a^	5.75 ± 0.33 ^e^	9.61 ± 3.96 ^b^	2.91 ± 0.38 ^fg^	5.68 ± 0.96 ^g^	4.10 ± 1.69 ^ef^	1.77 ± 0.35 ^bc^	2.72 ± 22 ^de^	0.88 ± 0.54 ^ab^	0.33 ± 0.05 ^abc^	6.99 ± 1.12 ^c^	3.45 ± 0.19 ^bcd^
2.	2.08 ± 0.03 ^c^	1.45 ± 0.22 ^a^	9.53 ± 2.37 ^cd^	2.31 ± 0.45 ^cdef^	1.11 ± 0.43 ^a^	9.05 ± 1.33 ^ij^	1.07 ± 0.54 ^a^	1.54 ±0.21 ^ab^	0.93 ± 0.05 ^ab^	0.62 ± 0.08 ^bcd^	7.64 ± 2.22 ^efg^	3.04 ± 0.09 ^b^
3.	0.75 ± 0.31 ^a^	1.81 ± 0.59 ^a^	11.43 ± 2.72 ^h^	4.45 ± 0.82 ^h^	3.19 ± 3.48 ^e^	1.01 ± 0.16 ^a^	1.28 ± 0.26 ^ab^	1.60 ± 0.69 ^ab^	0.89 ± 0.44 ^ab^	0.23 ± 0.04 ^a^	6.95 ± 1.34 ^cde^	3.47 ± 1.79 ^bcd^
4	5.94 ± 1.05 ^f^	2.10 ± 0.11 ^ab^	9.96 ± 1.04 ^de^	1.79 ± 0.28 ^abcd^	2.90 ± 0.54 ^de^	2.18 ± 0.83 ^bcd^	1.23 ± 0.19 ^ab^	1.74 ± 0.28 ^ab^	0.99 ± 0.34 ^ab^	0.21 ± 0.01 ^a^	6.97 ± 1.94 ^cde^	2.85 ± 0.88 ^b^
5	7.61 ± 1.53 ^i^	2.57 ± 1.53 ^b^	10.44 ± 1.86 ^ef^	1.41 ± 0.41 ^ab^	5.87 ± 0.26 ^g^	4.65 ± 0.44 ^fg^	1.07 ± 0.31 ^ab^	1.88 ± 0.65 ^ab^	1.04 ± 0.67 ^ab^	0.25 ± 0.01 ^a^	6.53 ± 1.48 ^c^	3.07 ± 0.73 ^b^
6.	0.86 ± 0.09 ^a^	2.58 ± 0.53 ^bc^	12.53 ± 3.54 ^i^	1.31 ± 0.89 ^a^	2.63 ± 0.15 ^cde^	2.71 ± 0.23 ^d^	1.32 ± 0.41 ^ab^	1.72 ± 0.74 ^ab^	0.92 ±0.25 ^ab^	0.27 ± 0.04 ^a^	6.96 ± 2.96 ^cde^	3.32 ± 0.65 ^bc^
7.	7.69 ± 0.25 ^i^	6.59 ±1.65 ^f^	16.26 ± 4.02 ^k^	4.71 ± 1.45 ^hi^	4.68 ± 0.66 ^f^	5.85 ± 0.61 ^h^	9.25 ± 2.94 ^i^	1.73 ± 0.23 ^ab^	2.20 ± 0.45 ^d^	0.44 ± 0.05 ^abc^	7.49 ± 1.72 ^def^	4.11 ± 1.38 ^de^
8.	1.54 ± 0.67 ^b^	1.70 ± 0.84 ^a^	16.93 ± 3.67 ^l^	2.16 ± 0.60 ^bcde^	2.41 ± 1.04 ^cd^	4.96 ± 1.91 ^g^	2.21 ± 0.56 ^cd^	4.05 ± 0.44 ^f^	1.12 ± 0.36 ^ab^	0.33 ± 0.03 ^ab^	8.32 ± 2.31 ^g^	4.39 ± 0.86 ^ef^
9.	3.27 ± 0.62 ^d^	3.30 ± 1.55 ^c^	10.72 ± 1.05 ^fg^	3.45 ± 0.39 ^g^	2.72 ± 0.13 ^cde^	10.42 ± 2.8 ^k^	5.75 ± 0.94 ^f^	1.59 ± 0.81 ^ab^	1.43 ± 0.88 ^abc^	0.28 ± 0.03 ^a^	7.02 ± 2.88 ^cde^	3.87 ± 1.48 ^cde^
10.	10.08 ± 3.78 ^k^	8.23 ± 1.32 ^h^	20.54 ± 1.43 ^m^	3.54 ± 0.23 ^g^	8.52 ± 1.02 ^j^	9.54 ± 3.02 ^j^	9.58 ± 3.92 ^i^	3.86 ± 0.67 ^f^	1.86 ± 0.96 ^cd^	0.85 ± 0.04 ^d^	8.24 ± 2.11 ^fg^	4.85 ± 1.12 ^f^
11.	9.31 ± 1.34 ^j^	15.08 ± 3.56 ^l^	16.05 ± 2.75 ^k^	5.46 ± 1.97 ^j^	12.36 ± 1.87 ^k^	13.84 ± 2.87 ^l^	8.21 ± 2.57 ^h^	5.53 ± 1.86 ^g^	3.85 ± 1.23 ^e^	1.81 ± 0.08 ^e^	9.51 ± 2.17 ^h^	10.28 ± 3.24 ^j^
12.	12.85 ± 2.99 ^l^	10.34 ± 2.87 ^j^	14.25 ± 2.54 ^j^	7.82 ± 1.76 ^k^	15.84 ± 2.98 ^l^	8.54 ± 1.57 ^i^	8.43 ± 2.68 ^h^	3.12 ± 0.76 ^e^	2.21 ± 1.75 ^d^	0.76 ± 0.06 ^d^	7.63 ± 1.79 ^efg^	8.43 ± 1.46 ^i^
13.	7.65 ± 1.65 ^i^	9.52 ± 2.35 ^i^	10.53 ± 1.65 ^ef^	2.58 ± 0.54 ^ef^	6.75 ± 1.97 ^h^	3.56 ± 1.12 ^e^	5.52 ± 1.68 ^f^	2.51 ± 0.17 ^cd^	1.46 ± 0.64 ^bc^	0.65 ± 0.06 ^bcd^	6.82 ± 1.67 ^cd^	5.68 ± 1.83 ^f^
14.	6.51 ± 1.63 ^gh^	8.62 ± 1.76 ^h^	12.46 ± 4.75 ^i^	2.61 ± 0.64 ^ef^	7.68 ± 0.98 ^i^	5.23 ± 1.75 ^gh^	6.88 ± 1.54 ^g^	1.68 ± 0.27 ^ab^	1.82 ± 0.82 ^cd^	0.68 ± 0.03 ^cd^	6.66 ± 1.48 ^c^	7.82 ± 1.72 ^h^
15.	5.84 ± 0.46 ^f^	11.22 ± 1.82 ^k^	11.26 ± 3.18 ^gh^	2.43 ± 0.73 ^def^	8.38 ± 3.65 ^j^	8.93 ± 2.49 ^ij^	2.51 ± 0.42 ^d^	1.82 ± 0.51 ^ab^	1.13 ± 0.76 ^ab^	0.33 ± 0.04 ^ab^	8.14 ± 1.25 ^fg^	3.04 ± 0.93 ^b^
16.	4.61 ± 1.86 ^e^	7.36 ±1.54 ^g^	15.67 ± 5.23 ^k^	1.84 ± 0.17 ^abcd^	2.24 ± 0.39 ^bcd^	9.59 ± 2.28 ^j^	2.28 ± 0.25 ^cd^	1.70 ± 0.43 ^ab^	1.03 ± 0.52 ^ab^	0.36 ± 0.02 ^abc^	6.79 ± 2.47 ^cd^	3.38 ± 0.21^bc^
17.	4.96 ± 1.74 ^e^	5.76 ± 0.86 ^ef^	5.80 ± 1.54 ^a^	1.30 ± 0.23 ^a^	1.05 ± 0.23 ^a^	1.75 ± 0.65 ^bc^	1.06 ± 0.33 ^a^	1.82 ± 0.46 ^ab^	0.75 ± 0.26 ^ab^	0.20 ± 0.02 ^a^	5.31 ± 1.37 ^b^	1.43 ± 0.17 ^a^
18.	6.19 ± 1.65 ^fg^	3.23 ± 0.56 ^c^	8.93 ± 2.49 ^c^	1.36 ±0.57 ^a^	1.64 ± 0.09 ^ab^	1.68 ± 0.83 ^b^	2.06 ± 0.73 ^cd^	1.41 ± 0.46 ^ab^	0.87 ± 0.26 ^a^	0.14 ± 0.01 ^a^	6.53 ± 2.09 ^c^	2.81± 0.17 ^b^
19.	2.75 ± 0.76 ^d^	4.53 ± 1.75 ^d^	9.37 ± 2.24 ^cd^	1.69 ± 0.87 ^abc^	2.10± 0.12 ^bc^	2.04 ± 0.97 ^bcd^	1.73 ± 0.87 ^bc^	1.03 ± 0.54 ^a^	4.26 ± 0.65 ^e^	0.17 ± 0.03 ^a^	4.37 ± 1.65 ^a^	1.81 ± 0.48 ^a^
20.	6.84 ± 1.31 ^h^	5.24 ± 2.76 ^e^	13.78 ± 2.08 ^j^	5.28 ± 1.59 ^ij^	1.64 ± 0.18 ^ab^	2.42 ± 0.13 ^j^	4.23 ± 0.28 ^e^	1.90 ± 0.61 ^bc^	1.19 ± 0.71 ^ab^	0.25 ± 0.02 ^a^	8.02 ± 3.33 ^fg^	3.98 ± 1.37 ^cde^
Hydromethanolic extracts												
1.	7.03 ± 1.05 ^d^	0.55 ± 0.04 ^b^	0.06 ± 0.02 ^a^	0.07 ± 0.01 ^a^	ND	ND	2.22 ± 0.20 ^b^	ND	ND	ND	0.19 ± 0.04 ^abc^	0.39 ± 0.07 ^cd^
2.	8.77 ± 1.83 ^e^	0.16 ± 0.05 ^a^	0.11 ± 0.04 ^a^	0.08 ± 0.01 ^a^	ND	ND	2.60 ± 0.92 ^bcd^	ND	ND	ND	0.18 ± 0.08 ^abc^	0.33 ± 0.98 ^bcd^
3.	1.66 ± 0.63 ^b^	0.05 ± 0.01 ^a^	ND	0.16 ± 0.04 ^a^	ND	ND	2.35 ± 1.01 ^bc^	ND	ND	ND	ND	ND
4.	ND	ND	ND	ND	ND	ND	2.43 ± 0.78 ^bcd^	ND	ND	ND	ND	ND
5.	1.73 ± 0.23 ^b^	ND	ND	ND	ND	ND	ND	ND	ND	ND	0.15 ± 0.07 ^abc^	ND
6.	11.33 ± 0.62 ^g^	ND	ND	0.15 ± 0.05 ^a^	ND	ND	2.30 ± 0.52 ^bc^	ND	ND	ND	0.33 ± 0.03 ^c^	0.59 ± 0.05 ^e^
7.	0.50 ± 0.09 ^a^	0.10 ± 0.04 ^a^	0.05 ± 0.01 ^a^	0.26 ± 0.02 ^a^	ND	ND	2.32 ± 0.78 ^bc^	ND	ND	ND	0.05 ± 0.01 ^a^	0.56 ± 0.11 ^e^
8.	6.12 ± 1.08 ^c^	1.25 ± 0.11 ^c^	0.17 ± 0.02 ^a^	1.81 ± 0.08 ^c^	ND	ND	2.35 ± 0.62 ^bc^	ND	ND	ND	0.15 ± 0.03 ^abc^	0.46 ± 0.21 ^de^
9.	1.96 ± 0.26 ^b^	ND	ND	0.17 ± 0.03 ^a^	ND	ND	2.84 ± 0.89 ^def^	ND	ND	ND	0.32 ± 0.06 ^bc^	0.45 ± 0.43 ^de^
10.	13.61 ± 1.86 ^h^	3.42 ± 0.75 ^f^	11.26 ±0.38 ^e^	6.84 ± 1.03 ^e^	ND	ND	1.18 ± 0.22 ^a^	ND	ND	ND	0.88 ± 0.04 ^e^	0.55 ± 0.47 ^e^
11.	1.88 ± 0.78 ^b^	2.13 ± 0.54 ^e^	2.18 ± 0.29 ^b^	1.23 ± 0.39 ^b^	ND	ND	2.42 ± 0.96 ^bcd^	ND	ND	ND	2.70 ± 0.11 ^g^	0.19 ± 0.07 ^b^
12.	1.60 ± 0.62 ^b^	5.88 ± 0.86 ^h^	8.70 ± 1.86 ^c^	5.26 ± 0.83 ^d^	ND	ND	3.80 ± 0.97 ^g^	ND	ND	ND	1.32 ± 0.32 ^f^	0.04 ± 0.01 ^a^
13.	8.99 ± 2.75 ^ef^	4.74 ± 1.06 ^g^	9.03 ± 1.68 ^d^	9.61 ± 1.83 ^f^	ND	ND	ND	ND	ND	ND	0.63 ± 0.09 ^d^	3.13 ± 0.06 ^g^
14.	9.39 ± 1.79 ^f^	6.99 ± 1.54 ^i^	0.19 ± 0.28 ^a^	0.16 ± 0.39 ^a^	ND	ND	3.14 ± 1.08 ^f^	ND	ND	ND	ND	4.92 ± 0.67 ^h^
15.	1.74 ± 0.29 ^b^	0.30 ± 0.06 ^ab^	0.13 ± 0.01 ^a^	0.22 ± 0.09 ^a^	ND	ND	2.75 ± 0.68 ^cdef^	ND	ND	ND	0.10 ± 0.03 ^ab^	0.94 ± 0.11 ^f^
16.	ND	1.56 ± 0.95 ^d^	ND	0.24 ± 0.01 ^a^	ND	ND	2.53 ± 0.22 ^bcde^	ND	ND	ND	0.11 ± 0.03 ^abc^	0.55 ± 0.08 ^e^
17.	1.79 ± 0.76 ^b^	3.19 ± 0.92 ^f^	0.07 ± 0.01 ^a^	0.27 ± 0.03 ^a^	ND	ND	3.91 ± 0.43 ^g^	ND	ND	ND	0.05 ± 0.01 ^a^	0.33 ± 0.07 ^bcd^
18.	1.63 ± 0.87 ^b^	1.44 ± 0.36 ^cd^	0.08 ± 0.01 ^a^	0.18 ± 0.01 ^a^	ND	ND	2.45 ± 0.67 ^bcd^	ND	ND	ND	0.17 ± 0.05 ^abc^	0.25 ± 0.07 ^bc^
19.	1.55 ± 0.13 ^b^	1.92 ± 0.66 ^e^	0.16 ± 0.09 ^a^	0.22 ± 0.04 ^a^	ND	ND	2.96 ± 0.83 ^ef^	ND	ND	ND	0.03 ± 0.01 ^a^	0.44 ± 0.10 ^de^
20.	1.79 ± 0.21 ^b^	2.10 ± 0.95 ^e^	ND	0.18 ± 0.07 ^a^	ND	ND	2.70 ± 0.19 ^cdef^	ND	ND	ND	0.04 ± 0.01 ^a^	0.56 ± 0.06 ^e^

Means followed by the same letter within a column indicate no significant difference (*p* < 0.05) in Duncan test. ND: not detectable.

**Table 3:** Antimicrobial activity of water and hydromethanolic extracts of *R. canina* L. against tested bacterial strains.

	*S. aureus* ATCC 6538	MRSA 18532	MRSA 43300	*S. epidermidis* ATCC 14990	*S. pyogenes*	*E. coli* ATCC 8739	*P. aeruginosa ATCC 9027*
Water extracts							
3.	-	-	-	-	-	128	128
6.	64/128	-	-	-	-	256	64
8.	128	64/128	64/128	64/128	-	-	-
10.	64	-	128	64/128	64/128	256	32/64
11.	4/8	4/8	2	0.5	32/64	-	-
12.	16	4/8	8	2	32/64	64	64
13.	4/8	4	2	1	256	256	-
14.	8/16	8/16	8	8	32/64	-	128
16.	-	-	-	-	64	128/-	-
17.	64	-	-	-	-	32/64	-
20.	8/16	-	-	-	-	32	-
Hydromethanolic extracts							
3.	16	16	16/32	16/32	32/128	32/64	*-*
6.	16	32/64	-	-	-	128	16/32
8.	32	8	-	-	-	64	-
9.	16	16	16/32	64/128	128	64	-
10.	2/4	2/4	4	0.5/1	-	-	-
16.	8	64	-	16	32/32	-	64
17.	8/16	16/32	16	16/32	32/32	32/64	64
18.	8	16	8/32	32/64	-	-	128
20.	2	16	16	8	32/32	32	-

Arabic numbers in the first column denote commercial samples of *R. canina* L. listed in Table 4.

**Table 4:** Characteristics of *Rosa canina* L. samples used in this study.

No.	Sample Name/ Sample Name on the Package	Part of the Plant/Composition	Place of Origin (Geographic Coordinates)/Manufacture of Plant Material
1.	Rosa canina	fruits	Wilkosy Zalesie (54°1′22′′ N, 21°42′23′′ E)
2.	Rosa canina	fruits	Kolonia Rybacka (54°11′27′′ N, 21°47′26′′ E)
3.	Rosa canina	fruits	Kolonia Rybacka (54°11′2′′ N, 21°47′42′′ E)
4.	Rosa canina	fruits	Węgorzewo (54°12′46′′ N, 21°44′28′′ E)
5.	Rosa canina	fruits	Węgorzewo (54°12′4.07′′ N, 21°44′40′′ E)
6.	Rosa canina	fruits	Mierzeszyn (54°12′11′′ N, 18°24′58′′ E)
7.	Rosa canina	fruits	Tczew (54°4′35′′ N, 18°45′42′′ E)
8.	Rosa canina	fruits	Gdansk (54°21′50′′ N, 18°42′48′′ E)
9.	Rosa canina	fruits	Tczew (54°5′15′′ N, 18°46′14′′ E)
10.	Rosa canina	leaves	Tczew (54°4′35′′ N, 18°45′42′′ E)
11.	Rosa canina	leaves	Gdansk (54°21′50′′ N, 18°42′48′′ E)
12.	Rosa canina	leaves	Tczew (54°5′15′′ N, 18°46′14′′ E)
13.	Rosa canina	leaves	Kolonia Rybacka (54°11′27′′ N, 21°47′26′′ E)
14.	Rosa canina	leaves	Kolonia Rybacka ((54°11′2′′ N, 21°47′42′′ E)
15.	Rosa canina	fruits	Kawon, Gostyn
16.	Ecological tea of rose fruits	fruits (95%) with hibiscus (5%)	Dary Natury, Koryciny
17.	Fruits tea	fruits	Herbapol, Lublin
18.	Fruits tea	fruits	Malva, Gorzów
19.	Ecological fruits tea	fruits	Bifix, Gorki Male
20.	Fruit-herbs tea	fruits with hibiscus	Lord Nelson, Lidl

**Table 5:** Analytical data: retention time (R_t_), analytical wavelength (λ_max_), mass spectral data and validation parameters: linear equation, correlation coefficient (R^2^), limit of detection (LOD) and limit of quantification (LOQ) of the calibration curves for LC-DAD-ESI/MS method.

Analyzed Phenolic Compounds	R_t_ (min)	λ_max_ (nm)	Molecular Ion [M + H]^+^ (*m*/*z*)	Molecular Ion [M − H]^−^ (*m*/*z*)	Linear Equation	R^2^	LOD (μg/mL)	LOQ (μg/mL)
Gallic acid	22.181	207/269/229/767/775	171.00	169.00	y = 40191x − 224863	0.998	3.02	10.54
Protocatechuic acid	28.764	264/208/292/229/767	155.00	153.00	y = 61072x − 518952	0.999	3.98	11.94
Vanillic acid	48.116	264/207/290/229/767	169.00	167.00	y = 5461x − 30114	0.997	4.34	14.93
Chlorogenic acid	52.409	214/326/241/776/767	355.25	353.25	y = 6791x + 17551	0.997	5.66	16.89
Syringic acid	57.264	207/271/230/767/776	199.05	197.05	y = 56849x + 745899	0.994	4.56	13.37
*p*-Coumaric acid	68.629	322/213/233/767/775	165.00	163.00	y = 55241x + 65487	0.994	2.65	6.98
Ferulic acid	84.437	327/216/237/770/759	195.05	193.05	y = 78049x + 546609	0.993	4.89	14.02
Sinapic acid	94.848	332/219/236/770/759	225.10	223.10	y = 21901x + 248523	0.994	5.34	16.30
Rutin	116.662	216/255/353/767/655	611.55	609.55	y = 19293x + 434342	0.997	4.23	13.87
Rosmarinic acid	127.006	208/328/235/775/473	361.30	359.30	y = 8888x + 100771	0.998	5.12	17.34
Cinnamic acid	135.413	291/269/207/225/767	149.00	147.00	y = 141045x + 46338	0.996	3.12	11.98
Quercetin	154.623	216/254/365/775/754	303.20	301.20	y = 83132x + 53978	0.995	4.13	13.42
